# Effect of Wrist Angle on Median Nerve Appearance at the Proximal Carpal Tunnel

**DOI:** 10.1371/journal.pone.0117930

**Published:** 2015-02-06

**Authors:** Ping Yeap Loh, Satoshi Muraki

**Affiliations:** 1 Department of Human Science, Graduate School of Design, Kyushu University, Minami-ku, Fukuoka, Japan; 2 Department of Human Science, Faculty of Design, Kyushu University, Minami-ku, Fukuoka, Japan; Università di Trento, ITALY

## Abstract

This study investigated the effects of wrist angle, sex, and handedness on the changes in the median nerve cross-sectional area (MNCSA) and median nerve diameters, namely longitudinal diameter (D1) and vertical diameter (D2). Ultrasound examination was conducted to examine the median nerve at the proximal carpal tunnel in both dominant and nondominant hands of men (n = 27) and women (n = 26). A total of seven wrist angles were examined: neutral; 15°, 30°, and 45° extension; and 15°, 30°, and 45° flexion. Our results indicated sexual dimorphism and bilateral asymmetry of MNCSA, D1 and D2 measurements. MNCSA was significantly reduced when the wrist angle changed from neutral to flexion or extension positions. At flexion positions, D1 was significantly smaller than that at neutral. In contrast, at extension positions, D2 was significantly smaller than that at neutral. In conclusion, this study showed that MNCSA decreased as the wrist angle changed from neutral to flexion or extension positions in both dominant and nondominant hands of both sexes, whereas deformation of the median nerve differed between wrist flexion and extension.

## Introduction

Over the past few decades, computers have become an essential tool in the workplace. The increase in computer use in daily life has caused an increase in discomfort at the neck, shoulder, elbow, wrist, and hand due to repetitive and awkward joint positions [1]. Approximately 20% of computer users experience musculoskeletal disorders of the upper extremities, such as carpal tunnel syndrome (CTS) [2,3]. CTS—one of the most commonly reported work-related musculoskeletal disorders of the upper extremities—is a peripheral nerve compression syndrome affecting the median nerve at the wrist carpal tunnel region [4]. The etiology of CTS is multifactorial, with mechanical compression stress on the median nerve considered one of the relevant factors. Bonfiglioli et al. [5] suggested that biomechanical stress and long hours of intensive manual work without adequate rest might cause impairment of the median nerve. The median nerve contributes to cutaneous sensation of the hand and fingers, as well as innervation to multiple hand muscles that control fine motor manipulation, including pinch and grip. Thus, individuals with CTS demonstrate weakened grip and pinch strength and decreased thumb and finger dexterity, which can affect daily life and work-related activities [6].

The carpal tunnel is formed by carpal bones as the floor and the transverse carpal ligament as the roof; the edge of the retinaculum at the pisiform defines the proximal carpal tunnel [7,8]. The carpal tunnel is a confined space comprising the median nerve and a total of nine tendons, including the flexor pollicis longus, four flexor digitorum superficialis, and four flexor digitorum profundus. The median nerve is located beneath and near the transverse carpal ligament, and is vulnerable to compression stress from intratunnel pressure and the surrounding structures. The carpal tunnel pressure at wrist normal posture was less than 15 mm Hg and the carpal tunnel pressure is elevated in flexion and extension wrist posture among non-CTS participants [8]. Previous studies have shown that individual or combined finger and thumb dynamic movements impose compression stress on the median nerve due to gliding motion of the tendons, leading to deformation of the median nerve [9]. On the other hand, radiocarpal and midcarpal joints contribute to such wrist movements as flexion, extension, radial deviation, and ulnar deviation. Carpal tunnel volume is influenced by dynamic kinematic movements of the carpal bones during wrist flexion and extension motion [10].

Well-designed equipment and workspace are necessary for computer users to perform daily work in a good posture so as to reduce the risk of work-related musculoskeletal disorders. Poor ergonomics of the wrist during daily life and computer work causes varying degrees of compression over the carpal tunnel. Computer users show a various range of wrist flexion and extension angles during typing work; the most commonly observed angle is 20° wrist extension with 20° ulnar deviation [11]. Therefore, it is important to understand the relationship between wrist angle and compression stress on the median nerve at the carpal tunnel.

A previous feasibility study of 12 male participants showed significant reduction of the median nerve cross-sectional area (MNCSA) at wrist flexion and extension positions compared with that at neutral [12]. In view of the small sample size and inclusion of only male participants, the current study is an extension of the research by Loh et al.[12], which increases the sample size, includes both male and female participants, and examines the median nerve in both dominant and nondominant hands. Duncan et al. [13] described an ellipse formula to calculate MNCSA by using longitudinal and vertical diameters of the median nerve; however, it is unknown whether wrist angle affects the median nerve diameters.

The objective of this study was to investigate the effects of wrist angle, sex, and handedness on changes in MNCSA and median nerve diameters, namely longitudinal diameter (D1) and vertical diameter (D2).

## Materials and Methods

### Participants

This study was approved by the Ethics Committee of the Faculty of Design at Kyushu University. Fifty-three healthy participants were recruited (Table 1), and written consent was obtained. The inclusion criteria for participation were able and willing to complete informed consent and above 20 years old. The exclusion criteria for participation were CTS patients, diabetic patients, history of wrist surgery or wrist fracture. All participants were free of signs and symptoms of CTS, as indicated by screening tests including the Boston Carpal Tunnel Questionnaire, Phalen Test, and CTS Tinel Test [14–16]. The physical anthropology characteristic of participants such as height, weight and wrist circumference were presented in Table 1. Meanwhile, forty-six participants were right-hand dominant (male = 23, female = 23) and seven participants were left-hand dominant (male = 4, female = 3), as indicated by the Edinburgh Handedness Inventory [17].

**Table 1:** Demographic data of participants (n = 53).

		Male (n = 27)	Female (n = 26)
Age (years)		24.9 ± 2.8	24.5 ± 3.2
Height (cm)		171.4 ± 5.8	159.8 ± 5.2
Weight (kg)		68.4 ± 13.1	51.6 ± 7.8
BMI (kg/m^2^)		23.3 ± 4.2	20.3 ± 3.1
Wrist Circumference (mm)	Right	160.6 ± 7.5	144.9 ± 8.1
	Left	158.9 ± 7.0	142.5 ± 8.9
Handedness	Right hand dominant	23	23
	Left hand dominant	4	3

### Ultrasound examination

Ultrasound examinations were performed using the LOGIQ e ultrasound system with transducer 12L-RS with imaging frequency bandwidth is 5–13 MHz (GE Healthcare, USA). A 7.0-mm-thick sonar pad (Nippon BXI Inc., Tokyo, Japan) was used as a coupling medium to standardize coupling thickness among all participants. B-mode with 12 MHz was used during the examination, and depth resolution of the ultrasound beam was adjusted accordingly to obtain clear images of the median nerve at the proximal carpal tunnel. The ultrasound images were obtained by same examiner.

Participants were examined in a seated position with the forearm in supination and rested on an arm support on the table. Participants were instructed to relax the forearm, wrist, and fingers during the examination. Examiner performed ultrasound examination on the passive wrist angle. During wrist angle positioning, examiner hold the palm across the heads of metacarpals and applied gentle force to maintain the wrist angle.

The pisiform bonymark was used as a landmark to identify the proximal carpal tunnel. An L-shaped plastic frame was placed along the radius bone with the perpendicular point located at the pisiform; the frame served as a location marker for the ultrasound probe during the examination. Ultrasound examination was performed for both dominant and nondominant wrists, and three images were taken for each wrist position. The ultrasound probe was removed and repositioned the probe for each image taking. The examination sequence of wrist position was as follows: neutral (0°); 15°, 30°, and 45° extension; and 15°, 30°, and 45° flexion. A 180° wrist goniometer was used to determine wrist angle. The triquetrum was used as an axis point for the goniometer, while the static arm of the goniometer was placed parallel to the ulnar bone and the moveable arm was placed parallel to the fifth metacarpal bone.

**Fig 1:**
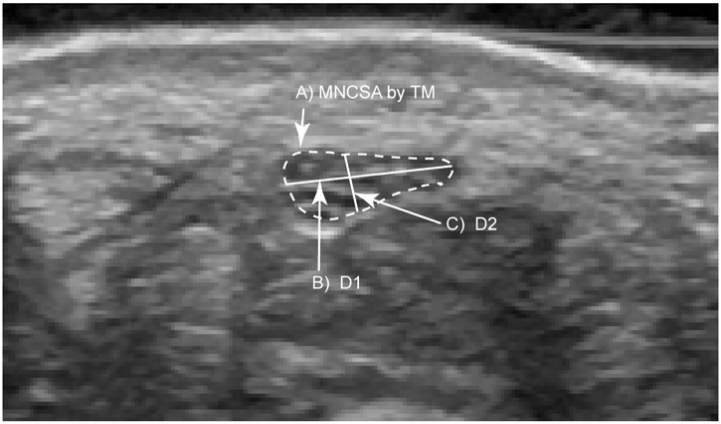
Ultrasound image of the median nerve at the proximal carpal tunnel. A) median nerve cross-sectional area by tracing method; B) longitudinal diameter; C) vertical diameter.

The median nerve was identified in the transverse plane across the proximal carpal tunnel. During ultrasound examination, the examiner identify the median nerve at the superficially level by a hypoechogenic rim which contained of the hypoechogenic nerve fascicles while the boundary of extraneural was recognized by hyperechogenic and thickened [18]. MNCSA was measured by a tracing method along the hypoechogenic boundary of the median nerve, and D1 and D2 were measured as described by Duncan et al. [13]. The D1 and D2 were identified by the longest perpendicular diameter of the median nerve. *ImageJ* [19] was used to calculate MNCSA, D1, and D2 (Fig. 1). Mean value of three images was calculated to represent the MNCSA, D1 and D2 at each wrist angles.

### Deformation percentage

Deformation percentages were calculated to determine differences in MNCSA, D1, and D2 at different wrist angles (15°, 30°, and 45° of both flexion and extension) compared with that at neutral. The following equation was used:

$$\text{deformation percentage}\;=\;\frac{\text{wrist neutral}-\text{different wrist angles}}{\text{wrist neutral}}\text{ × 100\%}$$

### Statistical analysis

Statistical analysis was performed using SPSS version 21.0 software (IBM Corporation, Chicago, IL). All results were expressed as mean ± SD. Inter- and intrarater reliability were calculated via reliability analysis in SPSS using 20 randomly selected ultrasound images. The Shapiro-Wilk normality test was conducted to examine the sample characteristics of MNCSA for both male and female participants in both dominant and nondominant hand group.

The paired *t* test was used to analyze differences in MNCSA, D1, and D2 at neutral position between dominant and nondominant hands in each male and female group. Subsequently, an independent samples *t* test was used to analyze differences in MNCSA, D1, and D2 at neutral position between male and female participants in each dominant and nondominant hand group.

Three-way repeated analysis of variance (2 x 7 x 2 factorial) was conducted with wrist side (dominant and nondominant), wrist flexion-extension positions (neutral; 15°, 30°, and 45° flexion; and 15°, 30°, and 45° extension), and sex as factors to examine difference in MNCSA at seven wrist positions. The assumption of sphericity was violated, as indicated by Mauchly’s test; therefore, Greenhouse-Geisser correction was used in the analysis of variance. Post-hoc pairwise Bonferroni-corrected comparison was used to examine mean differences in the factors.

## Results

### Inter- and intrarater reliability

Inter- and intrarater reliability for MNCSA, D1, and D2 measurements were good to excellent, according to Fleiss et al. [20] (Table 2).

**Table 2:** Inter- and intrarater reliability.

	Inter-rater reliability	Intra-rater reliability
MNCSA (mm^2^)	0.838	0.904
D1 (mm)	0.671	0.855
D2 (mm)	0.706	0.890

MNCSA = median nerve cross-sectional area

D1 = median nerve longitudinal diameter

D2 = median nerve vertical diameter

### Sample characteristics

The Shapiro-Wilk’s test (p > 0.05) [21,22] and visual inspection of histograms, normal Q-Q plots, and box plots showed that the MNCSAs were approximately normally distributed and slightly skewed and kurtotic (Table 3) for both male and female participants in both dominant and nondominant hands [23–25].

**Table 3:** Normality test for median nerve cross-sectional area.

	Wrist	Skewness (M ± SE)	Kurtosis (M ± SE)	Shapiro-Wilk Test (p value)
Male	Dominant	0.08 ± 0.45	0.53 ± 0.87	0.637
	Nondominant	-0.15 ± 0.45	-0.07 ± 0.87	0.816
Female	Dominant	0.01 ± 0.46	-1.04 ± 0.89	0.463
	Nondominant	0.06 ± 0.46	-0.89 ± 0.89	0.500

M = mean; SE = standard error

### Comparison of median nerve between dominant and nondominant hands at neutral

MNCSA, D1 and D2 of the dominant hand were significantly larger than those of the nondominant hand in both male and female participants (Table 4).

**Table 4:** Comparison between dominant and nondominant hands.

		Dominant hand	Nondominant hand	t	p
Male	MNCSA (mm^2^)	8.45 ± 1.15	7.41 ± 1.14	5.045	0.000
	D1 (mm)	4.99 ± 0.50	4.73 ± 0.48	2.070	0.052
	D2 (mm)	2.16 ± 0.19	2.00 ± 0.25	2.472	0.023
Female	MNCSA (mm^2^)	7.58 ± 1.01	6.58 ± 0.88	6.035	0.000
	D1 (mm)	4.88 ± 0.36	4.66 ± 0.44	2.080	0.053
	D2 (mm)	2.00 ± 0.25	1.82 ± 0.21	4.245	0.001

MNCSA = median nerve cross-sectional area

D1 = median nerve longitudinal diameter

D2 = median nerve vertical diameter

### Comparison of median nerve between male and female participants at neutral

MNCSA and D2 of male participants were significantly larger than those of female participants, but there was no significant difference in D1 between sexes in each group of dominant and nondominant hands (Table 5).

**Table 5:** Comparison between male and female participants.

		Male (n = 27)	Female (n = 26)	t	p
Dominant Hand	MNCSA (mm^2^)	8.45 ± 1.15	7.58 ± 1.01	2.478	0.018
	D1 (mm)	4.99 ± 0.50	4.88 ± 0.36	0.805	0.426
	D2 (mm)	2.16 ± 0.19	2.00 ± 0.25	2.238	0.031
Nondominant Hand	MNCSA (mm^2^)	7.41 ± 1.14	6.58 ± 0.88	2.497	0.017
	D1 (mm)	4.73 ± 0.48	4.66 ± 0.44	0.462	0.647
	D2 (mm)	2.00 ± 0.25	1.82 ± 0.21	2.380	0.023

MNCSA = median nerve cross-sectional area

D1 = median nerve longitudinal diameter

D2 = median nerve vertical diameter

### Change in MNCSA at different wrist angles

**Fig 2:**
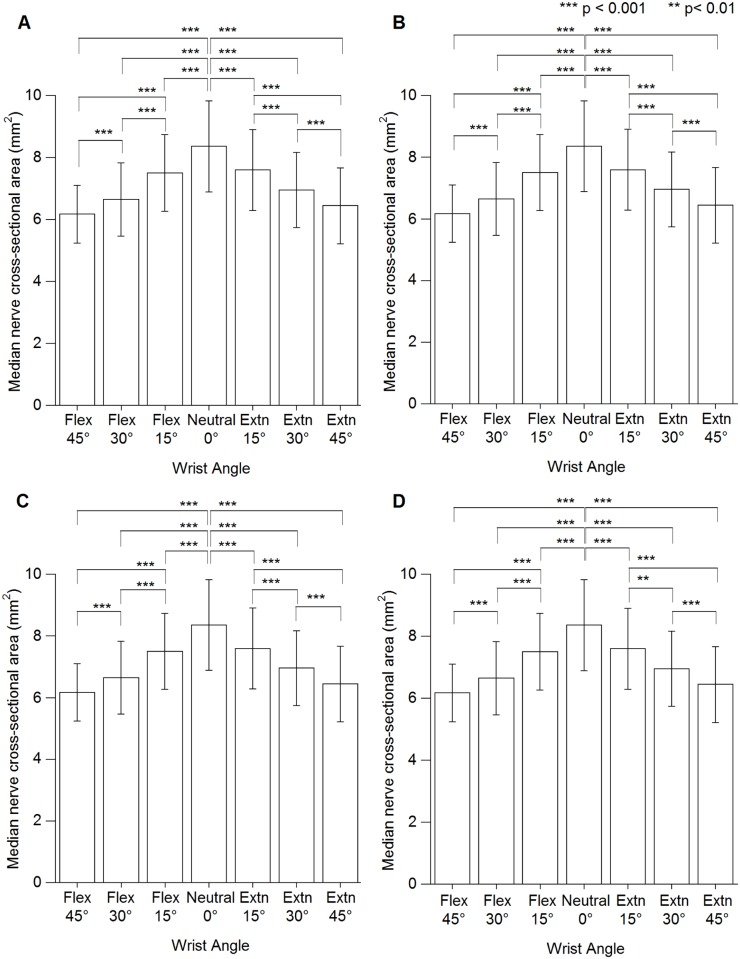
Median nerve cross-sectional area at different wrist angles. (A) Male dominant hand. (B) Male nondominant hand. (C) Female dominant hand. (D) Female nondominant hand. Extn = extension, Flex = flexion.

MNCSA at different wrist angles are presented in S1 Table. No significant interaction was found between wrist angle × handedness × sex (F [4.1, 208.8] = 1.355, p = 0.250). However, there was a significant interaction effect of wrist angle × handedness (F [4.1, 208.8] = 10.135, p < 0.001) and wrist angle × sex (F [3.4, 174.0] = 4.772, p < 0.01). For both male and female participants, wrist angle had a significant effect on MNCSA, which became smaller when the wrist changed from neutral to flexion or extension positions in both dominant and nondominant hands. A significant difference in MNCSA was found when comparing neutral position (0°) to 15°, 30°, and 45° flexion and to 15°, 30°, and 45° extension (Fig. 2).

### Changes in D1 and D2 at different wrist angles

**Fig 3:**
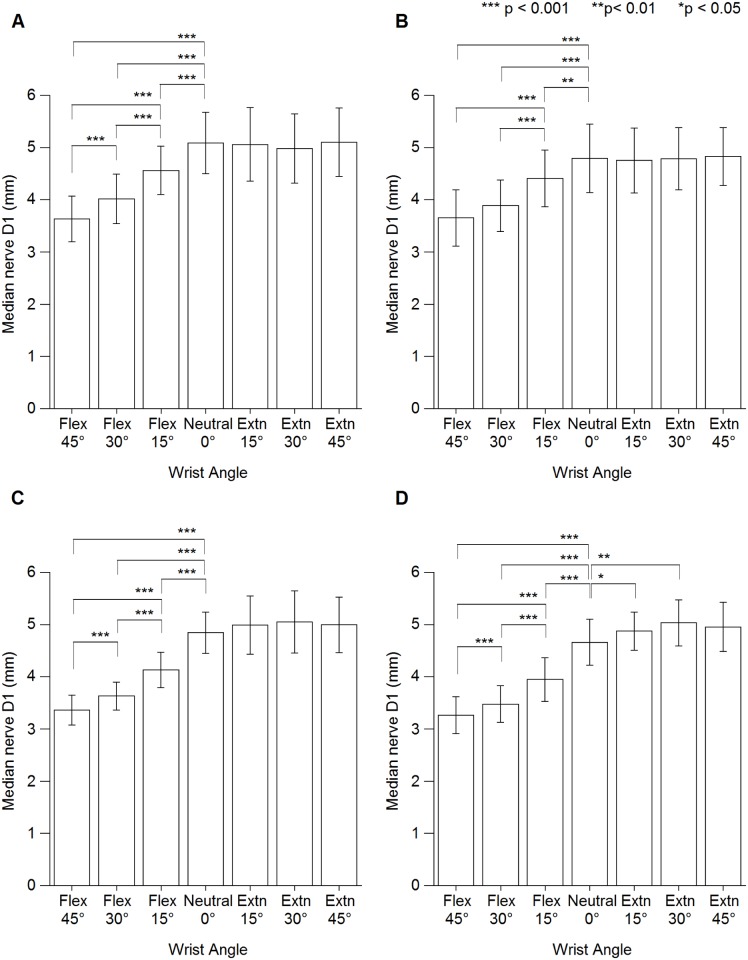
Median nerve longitudinal diameter (D1) at different wrist angles. (A) Male dominant hand. (B) Male nondominant hand. (C) Female dominant hand. (D) Female nondominant hand. Extn = extension, Flex = flexion.

**Fig 4:**
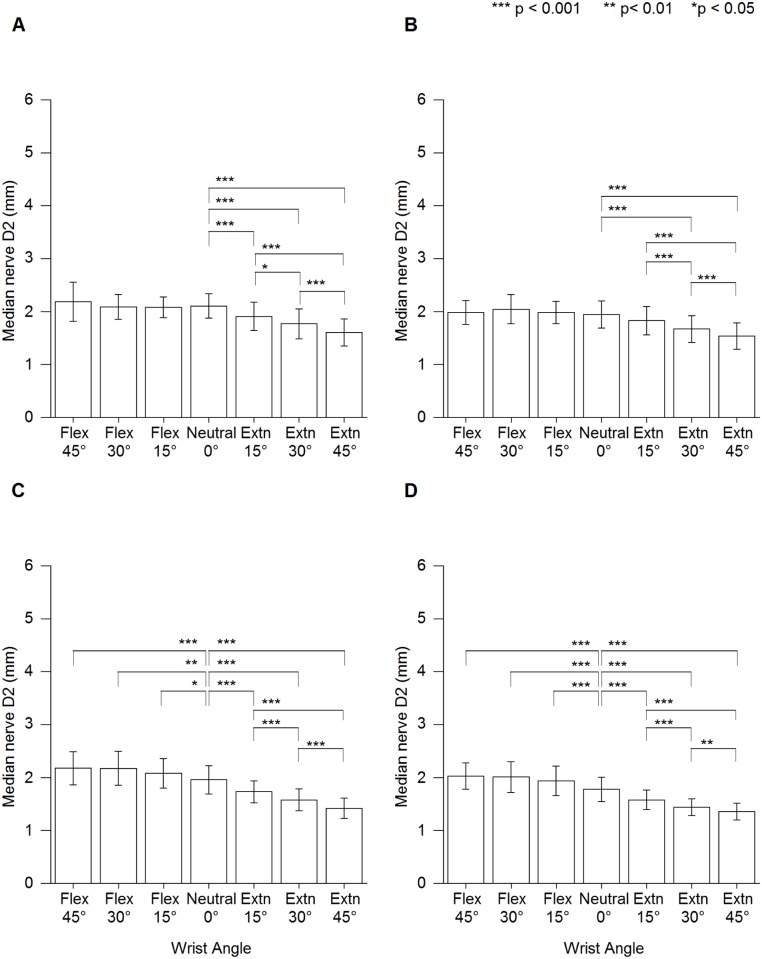
Median nerve vertical diameter (D2) at different wrist angles. (A) Male dominant hand. (B) Male nondominant hand. (C) Female dominant hand. (D) Female nondominant hand. Extn = extension, Flex = flexion.

Mean D1 and D2 at different wrist angles are presented in S2 and S3 Tables, respectively. The results showed no significant interaction between wrist angle × handedness × sex for both D1 and D2 (D1: F [4.0, 202.8] = 0.704, p = 0.590; D2: F [4.6, 233.6] = 1.313, p = 0.262). However, a significant interaction was found for the effect of wrist angle × handedness (D1: F [4.0, 202.8] = 2.429, p < 0.05; D2: F [4.6, 233.6] = 2.389, p < 0.05) and wrist angle × sex (D1: F [2.5, 146.0] = 7.817, p < 0.001; D2: F [2.532, 129.1] = 9.621, p < 0.001). Wrist flexion had a significant influence on D1, which became smaller compared with that at neutral for all participants. However, wrist 15° and 30° extension showed a significant increase in D1 at nondominant hand of female participants (Fig. 3). Comparatively, wrist flexion showed a significant increase in D2 compared with that neutral among female participants. However, wrist extension caused a significant decrease in D2 for all participants (Fig. 4).

### Deformation percentages of MNCSA, D1 and D2

Deformation percentage of the MNCSA at 15°, 30°, and 45° flexion were approximately-8%, -15%, and-21%, respectively, while that at 15°, 30°, and 45° extension were-7%, -14%, and-20%, respectively (Table 6). Furthermore, an increase of 15° in wrist flexion or extension caused significant reduction and higher deformation percentage of the MNCSA (Fig. 2 & Table 6).

**Table 6:** Deformation percentage of median nerve cross-sectional area at different wrist positions compared with at neutral.

MNCSA (mm^2^)		Flex 45°	Flex 30°	Flex 15°	Neutral	Extn 15°	Extn 30°	Extn 45°
Male	Dom	-25.6%	-20.2%	-11.8%	NA	-8.8%	-16.5%	-22.6%
	Non-Dom	-20.9%	-12.8%	-7.6%	NA	-6.5%	-14.0%	-19.1%
Female	Dom	-21.6%	-15.4%	-7.6%	NA	-8.1%	-14.9%	-23.4%
	Non-Dom	-16.9%	-12.0%	-5.1%	NA	-7.0%	-11.1%	-17.8%

Dom = dominant hand; Non-Dom = nondominant hand; Flex = flexion; Extn = extension; NA = Not applicable

At 15°, 30°, and 45° extension, both increase percentages of D1 and decrease percentages of D2 among female participants were higher than male participants (Tables 7 & 8). In contrast to wrist extension, at 15°, 30°, and 45° flexion, the decrease percentages of D1 and the increase percentages of D2 among female participants were higher than male participants (Tables 7 & 8).

**Table 7:** Deformation percentage of median nerve longitudinal diameter at different wrist positions compared with at neutral.

D1 (mm)		Flex 45°	Flex 30°	Flex 15°	Neutral	Extn 15°	Extn 30°	Extn 45°
Male	Dom	-28.2%	-20.6%	-11.6%	NA	-0.5%	-1.8%	0.7%
	Non-Dom	-23.3%	-18.4%	-8.9%	NA	-0.3%	0.6%	1.7%
Female	Dom	-30.2%	-24.6%	-17.7%	NA	3.2%	4.4%	3.5%
	Non-Dom	-28.0%	-23.4%	-16.1%	NA	4.4%	7.7%	6.2%

Dom = dominant hand; Non-Dom = nondominant hand; Flex = flexion; Extn = extension; NA = Not applicable

**Table 8:** Deformation percentage of median nerve vertical diameter at different wrist positions compared with at neutral.

D2 (mm)		Flex 45°	Flex 30°	Flex 15°	Neutral	Extn 15°	Extn 30°	Extn 45°
Male	Dom	4.1%	-0.3%	-1.6%	0.0%	-9.2%	-15.9%	-23.4%
	Non-Dom	2.6%	5.6%	1.5%	0.0%	-5.8%	-13.5%	-20.5%
	Female	Dom	11.7%	11.5%	5.6%	0.0%	-11.0%	-19.0%
Non-Dom	14.9%	13.5%	7.9%	0.0%	-10.8%	-18.5%	-23.2%	-27.0%

Dom = dominant hand; Non-Dom = nondominant hand; Flex = flexion; Ext = extension; NA = Not applicable

## Discussion

### Ultrasound imaging of the median nerve at carpal tunnel level

Various imaging techniques such as ultrasound, magnetic resonance imaging (MRI) and computerized tomography have been used in understand the carpal tunnel anatomy and the characteristic of median nerve among healthy person and CTS patients meanwhile [26–28]. MRI demonstrate high ability to observe the pathological changes of median nerve characteristic and the bowing of transverse carpal ligament among CTS patients [29–31]. Duymus et al. [26] found no significant differences of the MNCSA between both ultrasound and MRI measurement. The mean value of MNCSA at wrist neutral position in this study was close to the results reported in the MRI, which were 8.8 mm² at distal carpal tunnel and 10.0 mm² at proximal carpal tunnel [27]. Ultrasound imaging is one of the inexpensive and easy method to understand the behavior of median nerve under most circumstances such as real time evaluation during dynamic wrist and finger joints changes.

### Sex differences and bilateral asymmetry of the median nerve

It is well known that sex differences affect physical anthropological measurements, such as wrist circumference, arm perimeter, height, waist circumference, and body mass [32–35]. However, there has not been much research on sexual dimorphism of the upper limb peripheral nervous system. For one, the cross-sectional area of the ulnar nerve at the cubital tunnel in male adults is larger than that in female adults [36]. In this study, through ultrasound examination of the median nerve, our results showed that MNCSA and D2 of male participants were significantly larger than those of female participants (Table 5). Although D1 of men was larger than that of women, the difference was not significant. On the other hand, Moriyama [37] suggested no significant difference in peripheral nerves between men and women by microscopic examination of number of myelinated axons, average transverse area, and circularly ratios of myelinated axons. Based on cross-sectional area measurement, our results suggest sexual dimorphism of the median nerve at the proximal carpal tunnel.

Human upper limb anthropological measurements, such as arm length, elbow breadth, and hand bone sizes, show bilateral asymmetric features regardless of sex differences [38,39]. Our results showed significant bilateral asymmetry in MNCSA, D1 and D2 measurements in both male and female participants. Overall, our results of MNCSA, D1, and D2 measurements suggest bilateral asymmetric features of the median nerve.

### Relationship between wrist angle and MNCSA

Tendon excursion during active finger motion can cause deformation of the median nerve within the carpal tunnel [40]. However, the effect of passive wrist posture on the median nerve when the fingers are relaxed has not been clarified. During wrist motion, the contribution of radiocarpal and midcarpal joints in wrist flexion are 40% and 60%, respectively, while that in wrist extension are 66.5% and 33.5%, respectively [41]. The interaction between carpal bones and ligaments during flexion and extension causes the carpal bones to change position [42]; as a result, the relative locations of tendons and the median nerve at the carpal tunnel region may change. Therefore, we examined the effects of passive wrist flexion and extension angles on MNCSA.

From S1 Table, neutral position showed the largest MNCSA compared with that at all flexion and extension positions in both dominant and nondominant hands of both sexes. MNCSA decreased significantly as the wrist moved from neutral to extension or flexion positions. Mogk and Keir [28] reported that carpal tunnel volume was largest at neutral position compared with that at 30° flexion or extension. Carpal tunnel volume decreased by approximately 7.0% and 6.4% when the wrist moved from neutral to 30° extension and 30° flexion, respectively [10]. At 45° flexion or extension, deformation ratio was highest and MNCSA became smallest compared with that at other wrist angles. This could be due to carpal tunnel volume further decreasing as the wrist angle changed to 45° flexion or extension. Based on these results, it is important to keep the wrist near neutral position during daily work to prevent compression stress at the carpal tunnel.

### Relationships between wrist angle and D1 and D2

Peripheral nerves, such as the median nerve, show different unique characteristics in response to biomechanical stress, such as tensile stress and compression stress [43]. Biomechanical stress can be caused by joint angle, muscle contraction, and/or external compression force over the region [43]. The epineurium—the outermost layer of a peripheral nerve where there is loose connective tissue contained between the fascicles—allows the nerve to glide among the surrounding tissues, such as tendons. Consequently, the median nerve can be elongated and shortened during wrist extension and flexion, respectively. During extension, the median nerve is under strain stress, resulting in elongation into the palm area, while the surrounding tendons may impose shear force on the nerve [43]. The loose connective tissues are important for volume adaptation of the nerve during transverse compression stress [44]. Such volume adaptation also causes the shape of the MNCSA to change in response to biomechanical stress.

At wrist extension positions, the carpal tunnel pressure increased due to the incursion of finger flexor muscles into the proximal carpal tunnel which carpal tunnel volume decreased due to the flattening effect [8]. Furthermore, the transverse contraction stress and longitudinal tensile stress cause median nerve excursion and elongation [45]. Therefore, the biomechanical stress during passive wrist extension may cause the median nerve deformed in which the diameter increases longitudinally and decreases vertically, as found in this study.

During wrist flexion and finger flexion positions, the median nerve is exposes to the compression from the nine tendons within the carpal tunnel due to decrease carpal tunnel volume and the incursion of lumbrical muscles into the carpal tunnel as a result of finger flexion [8,10,28]. Carpal tunnel depth at wrist flexion the carpal tunnel depth is larger and the carpal tunnel width is smaller compare to wrist neutral [28]. The result of narrowed width of carpal tunnel may cause the decrease of longitudinal diameter of the median nerve due to the compression in longitudinal direction.

In all groups, wrist flexion positions showed a significant influence on D1, while wrist extension positions showed a significant influence on D2. Markedly, changes in D2 among female participants were significant at both wrist extension and flexion positions (Figs. 2 & 3) could be due to the female has smaller proximal carpal tunnel compare to male [8]. Therefore, change in D1 during wrist flexion and change in D2 during wrist extension can use as an indicator for median nerve compression during changes in wrist angle since D1 is more sensitive to wrist flexion and D2 is more sensitive to wrist extension.

CTS has a multifactorial etiology and mechanical compression stress on the median nerve is one of the relevant factors. Several studies reported that the pathophysiology of CTS involves a combination of mechanical and changes in synovial tissues within carpal tunnel [8,46,47]. The carpal tunnel pressure can be affected by both external and/or internal compression. External compression associated with such as low force for a long period, repetitive joint movements, direct contact pressure over volar wrist and vibration exposure [8,46,47]. On the other hand, the morphological and biochemical changes in synovial tissues such as transverse carpal ligament thickened, inflammation of tendon synovial sheath, edema and fibrosis [8,46].

Accumulated strain and stress over the wrist can lead to musculoskeletal disorders. Although we have not yet concluded the most suitable wrist angles to minimize stress on the median nerve during work, these findings could be applied in future research to investigate changes in the median nerve during specific work tasks. Furthermore, future research could be conducted on elderly individuals in the workplace. Better understanding of these changes will help to identify risk factors for musculoskeletal disorders and to apply preventive measures among computer users.

## Conclusion

The effect of wrist angle on deformation of the median nerve at the proximal carpal tunnel was investigated in this study. Our results showed that 15°, 30°, and 45° of wrist flexion or extension causes various changes in MNCSA and median nerve diameter measurements compared with those at neutral. It was shown that at wrist flexion positions, D1 became shorter and D2 became longer, whereas at extension positions, D1 became longer and D2 became shorter.

## Supporting Information

S1 Table Median nerve cross-sectional area (MNCSA) (mm^2^) at different wrist positions. (DOCX)

S2 Table Median nerve longitudinal diameter (D1) (mm) at different wrist positions. (DOCX)

S3 Table Median nerve vertical diameter (D2) (mm) at different wrist positions. (DOCX)
