# Supplementary material for: Effect of Wrist Angle on Median Nerve Appearance at the Proximal Carpal Tunnel
Source: PLoS One. 2015 Feb 6;10(2):e0117930. doi: 10.1371/journal.pone.0117930 (PMC4320094; doi:10.1371/journal.pone.0117930)
Supplement: S1 Table — (DOCX) [file pone.0117930.s001.docx]

**Table S1.** Median nerve cross-sectional area (MNCSA) (mm^2^) at different wrist positions.

|  | Male | | Female | |
| --- | --- | --- | --- | --- |
| Wrist Angle | Dominant | Nondominant | Dominant | Nondominant |
| Flexion 45° | 6.17 ± 0.93 | 5.73 ± 0.96 | 5.74 ± 0.81 | 5.31 ± 0.69 |
| Flexion 30° | 6.65 ± 1.18 | 6.34 ± 1.03 | 6.21 ± 0.95 | 5.64 ± 0.78 |
| Flexion 15° | 7.50 ± 1.23 | 6.79 ± 1.16 | 6.83 ± 0.94 | 6.10 ± 0.74 |
| Neutral (0°) | 8.36 ± 1.47 | 7.32 ± 1.40 | 7.34 ± 1.00 | 6.42 ± 0.85 |
| Extension 15° | 7.60 ± 1.31 | 6.85 ± 1.40 | 6.75 ± 1.00 | 5.97 ± 0.84 |
| Extension 30° | 6.96 ± 1.21 | 6.28 ± 1.21 | 6.25 ± 0.98 | 5.69 ± 0.74 |
| Extension 45° | 6.44 ± 1.22 | 5.88 ± 1.13 | 5.63 ± 0.90 | 5.26 ± 0.76 |
