# Supplementary material for: Effect of Wrist Angle on Median Nerve Appearance at the Proximal Carpal Tunnel
Source: PLoS One. 2015 Feb 6;10(2):e0117930. doi: 10.1371/journal.pone.0117930 (PMC4320094; doi:10.1371/journal.pone.0117930)
Supplement: S2 Table — (DOCX) [file pone.0117930.s002.docx]

**Table S2.** Median nerve longitudinal diameter (D1) (mm) at different wrist positions.

|  | Male | | Female | |
| --- | --- | --- | --- | --- |
| Wrist Angle | Dominant | Nondominant | Dominant | Nondominant |
| Flexion 45° | 3.63 ± 0.44 | 3.65 ± 0.54 | 3.36 ± 0.28 | 3.33 ± 0.32 |
| Flexion 30° | 4.02 ± 0.48 | 3.89 ± 0.49 | 3.63 ± 0.27 | 3.54 ± 0.32 |
| Flexion 15° | 4.56 ± 0.46 | 4.41 ± 0.54 | 4.13 ± 0.34 | 4.02 ± 0.40 |
| Neutral (0°) | 5.09 ± 0.59 | 4.79 ± 0.66 | 4.85 ± 0.40 | 4.65 ± 0.48 |
| Extension 15° | 5.06 ± 0.70 | 4.76 ± 0.62 | 4.99 ± 0.56 | 4.84 ± 0.38 |
| Extension 30° | 4.98 ± 0.67 | 4.79 ± 0.60 | 5.05 ± 0.60 | 4.99 ± 0.47 |
| Extension 45° | 5.11 ± 0.66 | 4.83 ± 0.55 | 5.00 ± 0.53 | 4.92 ± 0.44 |
