# Supplementary material for: Effect of Wrist Angle on Median Nerve Appearance at the Proximal Carpal Tunnel
Source: PLoS One. 2015 Feb 6;10(2):e0117930. doi: 10.1371/journal.pone.0117930 (PMC4320094; doi:10.1371/journal.pone.0117930)
Supplement: S3 Table — (DOCX) [file pone.0117930.s003.docx]

**Table S3.** Median nerve vertical diameter (D2) (mm) at different wrist positions.

|  | Male | | Female | |
| --- | --- | --- | --- | --- |
| Wrist Angle | Dominant | Nondominant | Dominant | Nondominant |
| Flexion 45° | 2.19 ± 0.37 | 1.98 ± 0.23 | 2.18 ± 0.31 | 2.03 ± 0.25 |
| Flexion 30° | 2.09 ± 0.23 | 2.05 ± 0.27 | 2.17 ± 0.32 | 2.01 ± 0.29 |
| Flexion 15° | 2.08 ± 0.20 | 1.98 ± 0.21 | 2.08 ± 0.28 | 1.94 ± 0.28 |
| Neutral (0°) | 2.11 ± 0.23 | 1.95 ± 0.26 | 1.96 ± 0.27 | 1.78 ± 0.23 |
| Extension 15° | 1.91 ± 0.27 | 1.83 ± 0.27 | 1.73 ± 0.21 | 1.58 ± 0.19 |
| Extension 30° | 1.77 ± 0.28 | 1.67 ± 0.25 | 1.58 ± 0.21 | 1.44 ± 0.16 |
| Extension 45° | 1.60 ± 0.26 | 1.54 ± 0.25 | 1.42 ± 0.19 | 1.36 ± 0.16 |
